# The Effect of Sorafenib, Tadalafil and Macitentan Treatments on Thyroxin-Induced Hemodynamic Changes and Cardiac Abnormalities

**DOI:** 10.1371/journal.pone.0153694

**Published:** 2016-04-15

**Authors:** Nancy S. Saad, Kyle Floyd, Amany A. E. Ahmed, Peter J. Mohler, Paul M. L. Janssen, Mohammad T. Elnakish

**Affiliations:** 1 Department of Physiology and Cell Biology, College of Medicine, The Ohio State University, Columbus, Ohio, United States of America; 2 Dorothy M. Davis Heart & Lung Research Institute, The Ohio State University, Columbus, Ohio, United States of America; 3 Department of Pharmacology and Toxicology, Faculty of Pharmacy, Helwan University, Cairo, Egypt; Scuola Superiore Sant'Anna, ITALY

## Abstract

Multikinase inhibitors (e.g. Sorafenib), phosphodiesterase-5 inhibitors (e.g. Tadalafil), and endothelin-1 receptor blockers (e.g. Macitentan) exert influential protection in a variety of animal models of cardiomyopathy; however, their effects on thyroxin-induced cardiomyopathy have never been investigated. The goal of the present study was to assess the functional impact of these drugs on thyroxin-induced hemodynamic changes, cardiac hypertrophy and associated altered responses of the contractile myocardium both *in-vivo* at the whole heart level and *ex-vivo* at the cardiac tissue level. Control and thyroxin (500 μg/kg/day)-treated mice with or without 2-week treatments of sorafenib (10 mg/kg/day; I.P), tadalafil (1 mg/kg/day; I.P or 4 mg/kg/day; oral), macitentan (30 and 100 mg/kg/day; oral), and their vehicles were studied. Blood pressure, echocardiography and electrocardiogram were non-invasively evaluated, followed by *ex-vivo* assessments of isolated multicellular cardiac preparations. Thyroxin increased blood pressure, resulted in cardiac hypertrophy and left ventricular dysfunction *in-vivo*. Also, it caused contractile abnormalities in right ventricular papillary muscles *ex-vivo*. None of the drug treatments were able to significantly attenuate theses hemodynamic changes or cardiac abnormalities in thyroxin-treated mice. We show here for the first time that multikinase (raf1/b, VEGFR, PDGFR), phosphodiesterase-5, and endothelin-1 pathways have no major role in thyroxin-induced hemodynamic changes and cardiac abnormalities. In particular, our data show that the involvement of endothelin-1 pathway in thyroxine-induced cardiac hypertrophy/dysfunction seems to be model-dependent and should be carefully interpreted.

## Introduction

Thyroid hormones [triiodothyronin (T3) and thyroxin (T4)] are known to have striking effects on the heart, ranging from physiologic cardiac hypertrophy with enhanced function [1] to cardiac dilation and heart failure [2]. Heart failure in both right ventricle (RV) and left ventricle (LV) has been reported in 6–15% of hyperthyroid patients [3, 4]. Timely and efficient treatment of cardiac manifestations in hyperthyroid patients is essential because cardiovascular complications comprise most of the deaths in these patients. The improvement of thyroid dysfunction must be the initial procedure applied in hyperthyroid patients with heart failure. Ultimate treatment of hyperthyroidism is frequently achieved to improve cardiac function [3, 4]; however, increased cardiac mortality has been shown as a trend in treated hyperthyroid patients [4, 5]. Therefore, the exact way to treat hyperthyroidism-associated heart failure remains incompletely understood and warrants further investigation.

Typically, hyperthyroidism-induced hypertrophy is more distinct in the RV than in the LV [6, 7]. Indeed, RV hypertrophy and RV dysfunction are key prognostic determinants of pulmonary artery hypertension (PAH) that is strongly linked to hyperthyroidism in both animals and humans [7–9]. Therefore, pharmacological treatments that target PAH and associated RV remodeling could be potential candidates for the treatment of marked RV remodeling in hyperthyroidism. Moreover, drugs that have been proven to improve both RV and LV remodeling may represent a standard therapy for hyperthyroidism-associated cardiomyopathy on the whole.

A number of growth factors that belong to transmembrane receptor tyrosine kinases such as platelet-derived growth factor (PDGF) and vascular endothelial growth factor (VEGF) have been shown to be involved in the abnormal cellular responses linked to pulmonary remodeling [10]. Experimental and clinical studies have revealed that PDGF inhibitors can decrease PAH [11, 12]. In addition, based on their role in vascular smooth muscle and myocardial hypertrophy serine/threonine kinases such as the Raf signaling pathway signify an attractive target for intervention in PAH [13, 14]. Contrasting to the majority of tyrosine kinases that are not expressed in the heart, serine/threonine kinases have been linked to myocardial hypertrophy. For instance, the Raf-1 has been verified as a key determinant of myocardial hypertrophy in mice following aortic banding [10, 14]. Recent reports have shown that the multikinase (raf1/b, VEGFR, PDGFR) inhibitor, sorafenib, can prevent pulmonary remodeling and improves cardiac function in experimental pulmonary hypertension. It has been concluded that combined inhibition of tyrosine and serine/threonine kinases may provide an option to treat PAH and associated RV remodeling [8, 10]. Beneficial effects of sorafenib on RV remodeling/dysfunction were attributed to the inhibition of Raf/MEK/ERK pathway [10], where ERK activation has been previously shown to be involved in the development of T4-induced cardiac hypertrophy [15].

The phosphodiesterase (PDE)-5 is a member of cyclic nucleotide PDE enzymes family that exclusively catalyzes cyclic guanosine monophosphate (cGMP), and its inhibition increases intracellular levels of cGMP [16]. PDE-5 inhibitors through targeting nitric oxide (NO)-regulated cGMP in penile vasculature result in smooth muscle relaxation, vasodilatation and increased blood flow [17]. Nonetheless, the functional impact of PDE-5 inhibition is not restricted to the human penis and it exists in different parts in the body, including pulmonary and systemic vasculature as well as hypertrophied myocardium [17]. Currently, PDE-5 inhibitors such as sildenafil and tadalafil are approved for PAH management [18]. Through their vasodilating effects on systemic and pulmonary blood vessels as well as their direct protecting effects on cardiomyocytes, PDE-5 inhibitors could be promising treatments for cardiovascular diseases [17]. In this regard, PDE-5 inhibitors demonstrated cardioprotective effects against cardiac remodeling, cardiac injury and LV failure [19–28] as well as PAH and RV failure both in animals [29] and humans [30]. Interestingly, PDE-5 inhibitors have been revealed to exert their protective effects through many signaling pathways, which are also common causative factors of T4-induced cardiac hypertrophy/dysfunction, such as oxidative stress, cardiomyocyte apoptosis, PI3K/Akt and ERK ½ [16, 17, 20, 31–34].

Endothelin-1 (ET-1) is a vasoactive peptide that works via activating 2 homologous G protein–coupled receptor subtypes, endothelin A (ET_A_) and endothelin B (ET_B_) [35]. ET-1 has been involved in the progression of heart failure. Plasma ET-1 levels as well as cardiac expression of ET-1 and its receptors, ET_A_ and ET_B_, are elevated in experimental animal models and in patients of heart failure [36–41]. Additionally, the Food Drug and Administration has approved the ET-1 receptor blockers for the treatment of PAH. Previous reports have proposed that a blockade of both ET_A_ and ET_B_ receptors is required to attain optimal efficacy [42]. Incidentally, dual ET-1 receptor antagonists such as bosentan and macitentan have been shown to improve symptoms and delay time to clinical worsening in PAH patients [43, 44]. Besides, they have been demonstrated to reverse PAH and RV remodeling in experimental animal models [35, 42, 45]. Importantly, previous reports have demonstrated that ET-1 contributes to cardiac hypertrophy and increased susceptibility to ischemia/reperfusion-induced ventricular fibrillation in the hyperthyroid myocardium [46–49].

Taking these findings into account, we sought to assess the functional impact of multikinase (raf1/b, VEGFR, PDGFR) inhibitor (Sorafenib), PDE-5 inhibitor (Tadalafil), and dual ET-1 receptor blocker (Macitentan) on the T4-induced cardiac hypertrophy and associated altered responses of the contractile myocardium both *in-vivo* at the whole heart level and *ex-vivo* at the cardiac tissue level using isolated papillary muscles from the RV of the mouse hearts.

## Methods

### Animals

Male FVB/N Mice (7–9 months old) were purchased from the Jackson Laboratory (ME, USA) and maintained at the Research Animal Facility of The Ohio State University. The experimental procedures and protocols used in this study were approved by the Animal Care and Use Committee of the Ohio State University, conforming to the Guide for the Care and Use of Laboratory Animals published by the United States National Institutes of Health (National Institutes of Health publication No. 85–23, revised 1996).

### Thyroxin (T4) and Drug Treatments

Sodium-L-thyroxin, T4, from Sigma-Aldrich (MO, USA) was prepared as previously described [50], and injected intraperitoneally at a once-daily dose of 500 μg/kg/day for two weeks [51, 52]. Sorafenib and tadalafil from Cayman Chemical (MI, USA) were dissolved in dimethyl sulfoxide (DMSO), freshly diluted with PBS (final DMSO concentration was 10%), and administered by intraperitoneal (I.P) injection. Sorafenib was administered at a dose of 10 mg/kg/day that has been shown to reverse PAH, RV remodeling and improve RV function in mice [8, 10]. Higher sorafenib doses, 30 mg/kg/day/I.P or 60 mg/kg/day/oral, have been shown to be cardiotoxic in cardiovascular-compromised mice [53] or result in excessive weight loss and death in nude mice [54], respectively. Thus, we did not go beyond the 10 mg/kg for this drug. On the other hand, tadalafil was used at a dose of 1 mg/kg/day/I.P (tadalafil_IP_) [20, 23, 55]. Also, tadalafil was suspended in PBS and administered by oral gavage in another group of mice at a dose of 4 mg/kg/day (tadalafil_Or_) [21, 22]. It was previously reported that both these doses/route of administration combinations were chosen based on the interspecies dose extrapolation scaling to result in plasma concentrations equivalent to a human dose of 20 mg/day and to be cardioprotective in mice [20–23, 55]. In addition, macitentan from Focus Synthesis LLC (CA, USA) was suspended in carboxymethylcellulose (CMC) solution [0.5% (wt/vol) carboxymethylcellulose sodium, 0.9% (wt/vol) NaCl, 0.4% (vol/vol) polysorbate, 0.9% (vol/vol) benzyl alcohol in deionized water] and administered by oral gavage at doses of 30 mg/kg/day (low dose: macitentan_LD_) and 100 mg/kg/day (high dose: macitentan_HD_). Although 10 mg/kg appeared to be the first maximal effective dose on hemodynamics for macitentan, previous studies used higher doses of 30 mg/kg and 100 mg/kg to ensure a positive effect on remodeling, and we decided to use the same high doses as described before [35, 42, 45]. Finally, all vehicles including the T4-vehicle (control), 10% DMSO and CMC solutions were administered by the same route of administration of their corresponding drugs for comparison. All drugs and vehicles were administered every day prior to T4 during the whole treatment period of the two weeks. A total of 141 mice were divided into 9 groups based on treatment as follows: Control: n = 22, T4: n = 34, DMSO: n = 14, Sorafenib: n = 13, Tadalafil_IP_: n = 22, Tadalafil_Or_: n = 8, CMC: n = 10, Macitentan_LD_: n = 10, Macitentan_HD_: n = 8.

At the end of the treatment period, animals underwent blood pressure, echocardiography and electrocardiogram analyses. Thereafter, animals were sacrificed; heart muscles were excised and processed for further *ex-vivo* experiments.

### Blood Pressure Measurements

Blood pressure was measured noninvasively in conscious untrained mice by the tail cuff method using a 6-Channel CODA High Throughput Acquisition system (Kent Scientific Corporation, Torrington, CT, USA) as previously described [51, 52]. Briefly, each experimental session consisted of 10 acclimatization cycles followed by 10 blood pressure measurements cycles. Only accepted cycles as identified by the blood pressure measurement software are included. The average of accepted cycles from one session was used for systolic, diastolic, and mean arterial blood pressure in each mouse.

### Echocardiography

*In-vivo* LV dimension and contractile function in mice were evaluated using a high-frequency ultrasound imaging system (VEVO 2100, Visual Sonics, Toronto, ON, Canada) as previously described [50–52] with minor changes. Experimental mice were anesthetized with isoflurane at a concentration of 3% and then maintained at 1.5% isoflurane using nasal prongs during the whole procedure. The measurements were taken from the parasternal short-axis view in M-mode to view the LV movement during systole and diastole corresponding to the electrocardiogram. All data and imaging were analyzed by the Visual Sonics Cardiac Measurements Package.

### Electrocardiogram (ECG)

ECG parameters including heart rate (HR), PR, QRS, and QT intervals were recorded noninvasively in conscious unrestrained mice using the ECGenie system (Mouse Specifics, Inc, MA) as we previously reported [51]. Mice were placed onto the recording platform for sufficient time (about 30 minutes) to acclimate and trigger recordings when their paws are in contact with the recording electrodes. All data were then analyzed by e-MOUSE, a Physiological Data Analysis and Database Portal.

### Heart Weight, Cardiac Muscle Preparation and Experimental Setup

First, mice were weighed, and then administered heparin by intraperitoneal injection. Five minutes later mice were euthanized by cervical dislocation. After bilateral thoracotomy, hearts were rapidly excised and placed in Krebs–Henseleit buffer containing (in mmol/L): 120 NaCl, 5 KCl, 2 MgSO_4_, 1.2 NaH2PO_4_, 20 NaHCO_3_, 0.25 Ca^2+^, and 10 glucose, equilibrated with 95% O_2_- 5% CO_2_, resulting in a pH of 7.4. Additionally, 20 mmol/L 2,3-butanedione monoxime (BDM) was added to the dissection buffer to prevent cutting injury [51, 52]. Non-cardiac tissues, such as pieces of lung, were carefully removed. Hearts were blotted gently on Kimwipes and then rapidly transferred to a small weigh dish. This dish contained clean oxygenated Krebs–Henseleit/BDM buffer that was tarred to zero on an electronic analytical balance to get the exact wet heart weight. Heart/body weight ratios were then calculated and expressed as mg/g. Hearts were then carefully opened, repeatedly perfused with the same oxygenated Krebs–Henseleit/BDM buffer, and blood was thoroughly washed out. Uniform linear papillary muscles were carefully dissected from the RV. The dimensions of muscles were measured using a calibration reticule in the ocular of the dissection microscope (40x, resolution ~ 10 μm). The cross-sectional areas were calculated assuming ellipsoid cross-sectional shapes. Average dimensions (width x thickness x length) were not significantly different compared to T4 (0.39 x 0.25 x 0.80 mm) as follows: Control (0.33 x 0.21 x 0.91 mm), DMSO (0.38 x 0.25 x 0.71 mm), sorafenib (0.31 x 0.20 x 0.82 mm), tadalafil_IP_ (0.38 x 0.26 x 0.79 mm), tadalafil_Or_ (0.32 x 0.21 x 0.91 mm), CMC (0.30 x 0.19 x 0.73 mm), macitentan_LD_ (0.30 x 0.19 x 0.58 mm) and macitentan_HD_ (0.31 x 0.20 x 0.75 mm). P values are 0.0929, 0.0829, and 0.4411, respectively.

With the use of the dissection microscope, muscles were mounted between basket-shaped extension of a force transducer (KG7, Scientific Instruments, Heidelberg, Germany) and a hook (valve end) connected to a micromanipulator as previously described [51, 52]. Muscles were superfused with the same buffer at 37.5°C as above (with the exception that BDM was omitted) and stimulated at 4 Hz. Extracellular Ca^2+^ concentration was raised to 2 mmol/L and muscles were allowed to stabilize for at least 30 minutes before the experimental protocol was initiated. As in our previous reports [51, 52], the 4 Hz baseline was selected rather than a more physiological 12 Hz. This was done in order to minimize run-down of the preparation [56]. However, to study more physiological frequencies, 12 Hz contractions were also assessed, but only for brief periods. Generally, muscles were stretched to an optimal length where a small increase in length resulted in nearly equal increases in resting tension and active developed tension. This length was selected to be comparable to the maximally attained length *in-vivo* at the end of diastole [57].

To obtain a broad scope of quantitative data to dissect contractile function and dysfunction, two of the three main mechanisms utilized *in-vivo* to physiologically modify force of contraction, frequency-dependent activation, and β-adrenergic stimulation were assessed in mouse papillary muscles under near physiological conditions as previously described [51, 52]. We assessed the effect of increasing stimulation frequencies between 4 and 14 Hz, spanning the entire *in-vivo* range of the mouse. At each frequency, forces were allowed to reach steady state before data were recorded. The effects of β-adrenergic stimulation were assessed by a concentration–response curve with isoproterenol (10^−9^–10^−6^ mol/l) at a baseline stimulation frequency of 4 Hz.

In all experiments performed peak isometric developed force (Fdev) was determined and normalized to the cross-sectional area of the muscle. Additionally, as a force-independent parameter of force decay kinetics, time to peak force (TTP), and time from peak force to 50% relaxation (RT50) were determined. Muscles of a Fdev of at least 5 mN/mm^2^ were only included in the analysis.

### Data Analysis and Statistics

Data are presented as mean ± SEM and were analyzed by one-way analysis of variance (ANOVA) followed by Dunnett Multiple Comparisons post-hoc test, comparing all groups to T4, and/or two-way ANOVA. A two-tailed value of P ≤ 0.05 was considered statistically significant.

## Results

At the end of the treatment period there was no significant difference in the body weights of all groups except for the sorafenib-treated mice that exhibited a significantly lower body weights compared to those of T4-treated mice (p < 0.01) (Table 1). Consistent with our previous findings [51, 52], heart weights and heart weight/body weight ratios as isolated parameters were significantly increased by T4 treatment compared to control (p < 0.01), confirming the development of cardiac hypertrophy in these mice. However, none of the drug treatments were able to attenuate these increases in the T4-treated mice (Table 1). Conversely, sorafenib resulted in a further increase in the heart weight/body weight ratio compared to T4-treated mice (p < 0.05), most probably due to significantly lower final body weights in these mice (Table 1).

**Table 1:** Morphological Data.

Group	BW (g)	HW (mg)	HW/BW (mg/g)
**Control**	28.3 ± 0.4	131.3 ± 1.7*	4.65 ± 0.05*
**T4**	28.9 ± 0.5	173.1 ± 2.8	6.02 ± 0.07
**DMSO + T4**	29.3 ± 0.7	179.4 ± 5.7	6.13 ± 0.15
**Sorafenib + T4**	25.6 ± 1.0*	163.7 ± 5.5	6.45 ± 0.17*
**Tadalafil**_**IP**_ **+ T4**	28.3 ± 0.5	176.1 ± 3.5	6.22 ± 0.08
**Tadalafil**_**Or**_ **+ T4**	29.8 ± 0.8	174.2 ± 6.0	5.84 ± 0.09
**CMC + T4**	28.6 ± 0.4	171.5 ± 6.5	6.01 ± 0.20
**Macitentan**_**LD**_ **+ T4**	27.1 ± 0.7	166.7 ± 3.4	6.16 ± 0.10
**Macitentan**_**HD**_ **+ T4**	28.8 ± 0.4	172.0 ± 2.2	5.98 ± 0.07

BW: body weight, HW: heart weight, Control; n = 21, Thyroxin (T4); n = 33, Dimethyl sulfoxide (DMSO); N = 14, Sorafenib; n = 13, Tadalafil_IP_ (intraperitoneal, 1 mg/kg); n = 21, Tadalafil_Or_ (oral, 4 mg/kg); n = 8, carboxymethylcellulose (CMC); n = 10, Macitentan_LD_ (Low dose: 30 mg/kg); n = 10, Macitentan_HD_ (High dose: 100 mg/kg); n = 8.

*: indicates a significant change as revealed by one-way ANOVA followed by Dunnett Multiple Comparisons post-hoc test, comparing all groups to T4.

**Fig 1:**
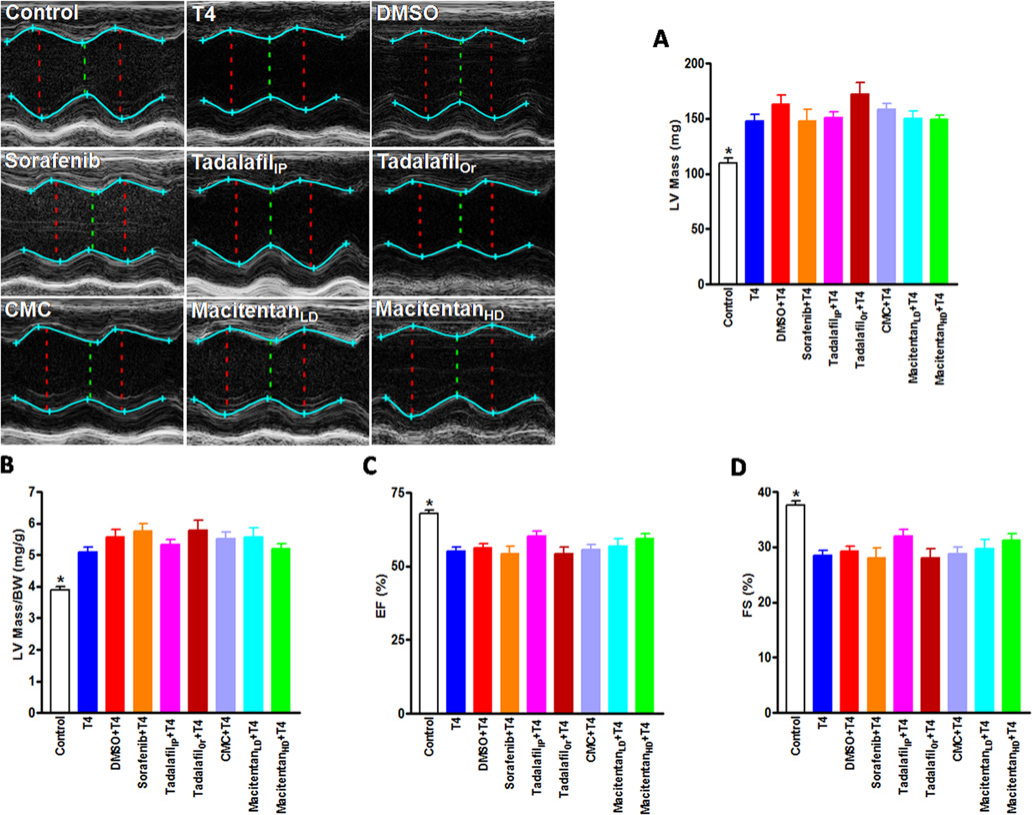
Echocardiography Analysis of the Mouse Hearts. (**A**) Representative bar graphs show left ventricular (LV) mass, (**B**) LV mass/body weight (BW) ratio, (**C**) ejection fraction (EF), and (**D**) fractional shortening (FS) in mice. Control; n = 22, Thyroxin (T4); n = 34, Dimethyl sulfoxide (DMSO); N = 14, Sorafenib; n = 13, Tadalafil_IP_ (intraperitoneal, 1 mg/kg); n = 22, Tadalafil_Or_ (oral, 4 mg/kg); n = 8, carboxymethylcellulose (CMC); n = 10, Macitentan_LD_ (Low dose: 30 mg/kg); n = 10, Macitentan_HD_ (High dose: 100 mg/kg); n = 8. *: indicates a significant change as revealed by one-way ANOVA followed by Dunnett Multiple Comparisons post-hoc test, comparing all groups to T4.

Assessment of cardiac dimensions by echocardiography revealed significant increases in both LV mass (148 ± 6 mg; p < 0.01) (Fig 1A) and LV mass/body weight ratio (5.10 ± 0.15 mg/g; p < 0.01) (Fig 1B) of the hearts of T4-treated mice compared to those of control (110 ± 4 mg, and 3.89 ± 0.12 mg/g, respectively), which confirms our morphological data. Again, none of the drug treatments were able to attenuate these increases in the LV mass (DMSO: 163 ± 8 mg, sorafenib: 148 ± 10 mg, tadalafil_IP_: 151 ± 5 mg, tadalafil_Or_: 172 ± 11 mg, CMC: 158 ± 6 mg, macitentan_LD_: 150 ± 7 mg and macitentan_HD_: 149 ± 4 mg) (Fig 1A) or in the LV mass/body weight ratio (DMSO: 5.57 ± 0.25 mg/g, sorafenib: 5.75 ± 0.25 mg/g, tadalafil_IP_: 5.33 ± 0.16 mg/g, tadalafil_Or_: 5.77 ± 0.34 mg/g, CMC: 5.52 ± 0.22 mg/g, macitentan_LD_: 5.57 ± 0.28 mg/g and macitentan_HD_: 5.19 ± 0.16 mg/g) (Fig 1B) of the hearts of T4-treated mice. In line with our previous data [51, 52], echocardiography analysis of the mouse hearts showed that LV systolic functions were compromised in the hearts of T4-treated mice as evident by significantly decreased ejection fraction (EF) (55.02 ± 1.41%; p < 0.01) (Fig 1C) and fractional shortening (FS) (28.50 ± 0.93%; p < 0.01) (Fig 1D) compared to those of control (EF: 68.05 ± 0.97% and FS: 37.53 ± 0.78%). Still, none of the drug treatments were able to improve the EF (DMSO: 56.32 ± 1.30%, sorafenib: 54.31 ± 2.37%, tadalafil_IP_: 60.10 ± 1.72%, tadalafil_Or_: 54.14 ± 2.41%, CMC: 55.62 ± 1.84%, macitentan_LD_: 56.87 ± 2.54% and macitentan_HD_: 59.33 ± 1.76%) (Fig 1C) or FS (DMSO: 29.24 ± 0.87%, sorafenib: 28.05 ± 1.73%, tadalafil_IP_: 32.04 ± 1.19%, tadalafil_Or_: 28.03 ± 1.60%, CMC: 28.81 ± 1.24%, macitentan_LD_: 29.71 ± 1.61% and macitentan_HD_: 31.28 ± 1.18%) (Fig 1D) compared to those of T4-treated mice.

In close agreement with our previous reports on such treatment [51, 52], T4-treated mice exhibited significant increases in systolic (p < 0.01), diastolic (p < 0.05) and mean arterial pressure (MAP) (p < 0.01) compared to those in control. Yet, none of the drug treatments were able to reverse these increases in the T4-treated mice (Table 2).

**Table 2:** Blood Pressure of Mice.

Group	SBP (mmHg)	DBP (mmHg)	MAP (mmHg)
**Control**	122 ± 7*	93 ± 7*	101 ± 7*
**T4**	144 ± 3	109 ± 3	120 ± 3
**DMSO + T4**	147 ± 4	116 ± 4	126 ± 4
**Sorafenib + T4**	149 ± 4	112 ± 5	124 ± 5
**Tadalafil**_**IP**_ **+ T4**	144 ± 2	111 ± 2	122 ± 2
**Tadalafil**_**Or**_ **+ T4**	132 ± 4	103 ± 4	112 ± 4
**CMC + T4**	151 ± 2	119 ± 3	130 ± 3
**Macitentan**_**LD**_ **+ T4**	148 ± 4	116 ± 4	126 ± 4
**Macitentan**_**HD**_ **+ T4**	149 ± 2	121 ± 2	130 ± 2

SBP: systolic blood pressure, DBP: diastolic blood pressure, MAP: mean arterial pressure, Control; n = 11, Thyroxin (T4); n = 22, Dimethyl sulfoxide (DMSO); N = 6, Sorafenib; n = 13, Tadalafil_IP_ (intraperitoneal, 1 mg/kg); n = 7, Tadalafil_Or_ (oral, 4 mg/kg); n = 8, carboxymethylcellulose (CMC); n = 10, Macitentan_LD_ (Low dose: 30 mg/kg); n = 10, Macitentan_HD_ (High dose: 100 mg/kg); n = 8.

*: indicates a significant change as revealed by one-way ANOVA followed by Dunnett Multiple Comparisons post-hoc test, comparing all groups to T4.

On the other hand, investigating the electrical activity of the mouse hearts using the ECG analysis demonstrated similar values for PR, QRS, and QT intervals in both control and T4-treated mice, and it did not reveal any sign of arrhythmia as we reported before [51]. Besides, none of the drug treatments affected these parameters or resulted in any abnormalities in the cardiac electrical activity following T4 treatment (Table 3).

**Table 3:** Electrocardiogram Analysis of Mice.

Group	PR (ms)	QRS (ms)	QT (ms)
**Control**	24 ± 1	11 ± 0.4	44 ± 1
**T4**	24 ± 1	11 ± 1	42 ± 1
**DMSO + T4**	23 ± 2	10 ± 1	42 ± 2
**Sorafenib + T4**	24 ± 1	11 ± 1	42 ± 1
**Tadalafil**_**IP**_ **+ T4**	23 ± 1	12 ± 0.4	42 ± 1
**Tadalafil**_**Or**_ **+ T4**	21 ± 1	10 ± 1	41 ± 1
**CMC + T4**	24 ± 1	10 ± 1	42 ± 1
**Macitentan**_**LD**_ **+ T4**	23 ± 1	10 ± 1	43 ± 2
**Macitentan**_**HD**_ **+ T4**	21 ± 2	11 ± 1	43 ± 2

ms: millisecond, Control; n = 7, Thyroxin (T4); n = 12, Dimethyl sulfoxide (DMSO); N = 6, Sorafenib; n = 9, Tadalafil_IP_ (intraperitoneal, 1 mg/kg); n = 7, Tadalafil_Or_ (oral, 4 mg/kg); n = 8, carboxymethylcellulose (CMC); n = 10, Macitentan_LD_ (Low dose: 30 mg/kg); n = 9, Macitentan_HD_ (High dose: 100 mg/kg); n = 7.

In the current study, mouse HR values have been obtained from 3 different assessments, including the tail cuff, echocardiography and electrocardiogram. HR of the conscious restrained and unrestrained mice using tail cuff and electrocardiogram, respectively, revealed no significant difference between T4-treated mice and all other treatments (Table 4). Conversely, echocardiographic analysis of anaesthetized mice showed a significant increase in the HR of T4-treated mice compared to control (p < 0.01). However, none of the drug treatments were able to decrease such increase (Table 4).

**Table 4:** Heart Rate Analysis of Mice.

Heart Rate (BPM)			
Group	Tail Cuff	Echocardiography	Electrocardiogram
**Control**	742 ± 16	476 ± 9*	698 ± 22
**T4**	702 ± 13	575 ± 9	707 ± 22
**DMSO + T4**	706 ± 15	556 ± 14	669 ± 19
**Sorafenib + T4**	699 ± 13	600 ± 9	711 ± 15
**Tadalafil**_**IP**_ **+ T4**	712 ± 14	551 ± 10	717 ± 18
**Tadalafil**_**Or**_ **+ T4**	707 ± 17	611 ± 14	706 ± 7
**CMC + T4**	685 ± 12	592 ± 9	728 ± 16
**Macitentan**_**LD**_ **+ T4**	669 ± 20	568 ± 8	705 ± 26
**Macitentan**_**HD**_ **+ T4**	661 ± 14	585 ± 8	697 ± 11

BPM: beat per minute. Tail Cuff: Control; n = 10, Thyroxin (T4); n = 21, Dimethyl sulfoxide (DMSO); N = 6, Sorafenib; n = 13, Tadalafil_IP_ (intraperitoneal, 1 mg/kg); n = 7, Tadalafil_Or_ (oral, 4 mg/kg); n = 8, carboxymethylcellulose (CMC); n = 10, Macitentan_LD_ (Low dose: 30 mg/kg); n = 10, Macitentan_HD_ (High dose: 100 mg/kg); n = 8. Echocardiography: Control; n = 22, T4; n = 34, DMSO; N = 14, Sorafenib; n = 13, Tadalafil_IP_; n = 22, Tadalafil_Or_; n = 8, CMC; n = 10, Macitentan_LD_; n = 10, Macitentan_HD_; n = 8. Electrocardiogram Analysis: Control; n = 7, T4; n = 12, DMSO; N = 6, Sorafenib; n = 9, Tadalafil_IP_; n = 7, Tadalafil_Or_; n = 8, CMC; n = 10, Macitentan_LD_; n = 9, Macitentan_HD_; n = 7.

*: indicates a significant change as revealed by one-way ANOVA followed by Dunnett Multiple Comparisons post-hoc test, comparing all groups to T4.

Physiological changes in cardiac contractile strength are mainly governed via three mechanisms: length-dependent activation (Frank-Starling mechanism), frequency-dependent activation (Bowditch effect), and adrenergic stimulation (fight/flight response). To characterize potential deficiencies in cardiac contractile strength, we tested the contractile performance on papillary muscles isolated from the RV of the mouse hearts while only varying frequency-dependent activation and adrenergic stimulation because the length-dependent activation is preserved in the hearts of these T4-treated mice as we have recently described [52]. Similar to our previous results [51], Fdev of papillary muscles from the RV of T4-treated mouse hearts was not statistically significant from those of the control mice under near physiological temperature and at a preload resulting in sarcomere length around the *in-vivo* end-diastolic values of 2.2 μm [57]. The Fdev was also maintained in all other groups except for the tadalafil_Or_ and the CMC groups which unexpectedly showed significantly higher Fdev values (p < 0.05 and p < 0.01, respectively) compared to T4 group (Table 5). In contrast, muscles from T4-treated mice contracted and relaxed more rapidly compared to those from control mice as indicated by significantly decreased TTP (p < 0.01) and RT50 (p < 0.01). None of the drug treatments were able to significantly change the TTP or the RT50 following T4 treatment except for the sorafenib, which only returned the RT50 close to control value (p < 0.05) (Table 5).

**Table 5:** Contractile Profile of Isolated Right Ventricular Papillary Muscles.

Group	Fdev (mN/mm^2^)	TTP (ms)	RT50 (ms)
**Control**	14 ± 2	49 ± 1*	23 ± 1*
**T4**	11 ± 1	41 ± 1	18 ± 1
**DMSO + T4**	14 ± 2	38 ± 1	18 ± 1
**Sorafenib + T4**	15 ± 4	44 ± 4	22 ± 2*
**Tadalafil**_**IP**_ **+ T4**	12 ± 2	41 ± 1	17 ± 1
**Tadalafil**_**Or**_ **+ T4**	21 ± 3*	41 ± 1	16 ± 0.4
**CMC + T4**	24 ± 5*	39 ± 1	17 ± 1
**Macitentan**_**LD**_ **+ T4**	16 ± 3	39 ± 1	16 ± 1
**Macitentan**_**HD**_ **+ T4**	19 ± 3	38 ± 1	16 ± 1

Fdev: isometric developed force, TTP: time to peak, RT50: 50% relaxation time, Control; n = 12, Thyroxin (T4); n = 15, Dimethyl sulfoxide (DMSO); N = 10, Sorafenib; n = 9, Tadalafil_IP_ (intraperitoneal, 1 mg/kg); n = 10, Tadalafil_Or_ (oral, 4 mg/kg); n = 8, carboxymethylcellulose (CMC); n = 8, Macitentan_LD_ (Low dose: 30 mg/kg); n = 8, Macitentan_HD_ (High dose: 100 mg/kg); n = 7.

*: indicates a significant change as revealed by one-way ANOVA followed by Dunnett Multiple Comparisons post-hoc test, comparing all groups to T4.

**Fig 2:**
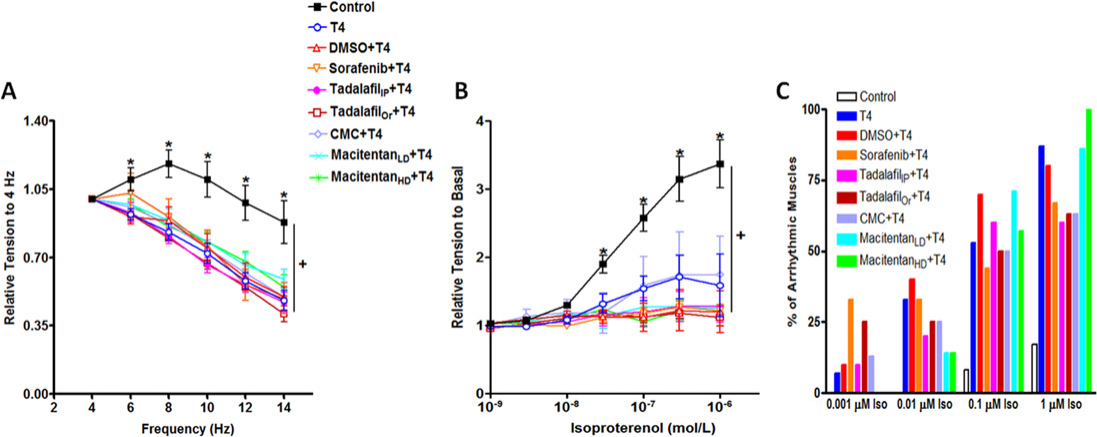
Physiological Modification of the Contractile Force of Isolated Right Ventricular Papillary Muscles. **(A) Frequency-dependent activation**; Isometric developed force values are expressed as a fraction of its corresponding value at the basal frequency of 4 Hz and presented as mean ± SEM, and (**B**) **β–adrenergic stimulation**; Isometric developed force values are expressed as a fraction of its corresponding value at the basal frequency of 4 Hz before isoproterenol addition and presented as mean ± SEM. Control; n = 12, Thyroxin (T4); n = 15, Dimethyl sulfoxide (DMSO); N = 10, Sorafenib; n = 9, Tadalafil_IP_ (intraperitoneal, 1 mg/kg); n = 10, Tadalafil_Or_ (oral, 4 mg/kg); n = 8, carboxymethylcellulose (CMC); n = 8, Macitentan_LD_ (Low dose: 30 mg/kg); n = 8, Macitentan_HD_ (High dose: 100 mg/kg); n = 7. Note: in the β–adrenergic stimulation curve (**B**), all isometric developed force values at which the muscles exhibited an arrhythmic behavior were excluded from the analysis. For example, the Macitentan_HD_ group has no representative point at isoproterenol concentration of 1 μM, because all muscles became arrhythmic at this concentration [i.e. 7 out of 7 (100%)]. (**C**) **Development of Arrhythmia**: % of arrhythmic muscles at different isoproterenol (Iso) concentrations. The absence of the representative bar of any group at any Iso concentration on the curve means the absence of arrhythmia at this concentration. *: indicates a significant change as revealed by one-way ANOVA followed by Dunnett Multiple Comparisons post-hoc test, comparing all groups to T4. +: indicates a significant change as revealed by two-way ANOVA.

Fdev was determined not only at the baseline frequency of stimulation of 4 Hz but also within the murine *in-vivo* physiological range (8–12 Hz), thereby allowing for a less ambiguous extrapolation to *in-vivo* outcome. Two-way ANOVA showed that at least one of the drugs/frequencies differs from the others with respect to relative tension change (p = 0.000). Repeated one-way ANOVA at each frequency clearly showed that muscles from control mice positively responded to increasing frequencies signifying a positive force-frequency relationship (FFR); however, muscles from T4-treated mice showed significantly (p < 0.05 at 6 Hz, and p < 0.01 at all other frequencies) lower changes in the Fdev at all tested frequencies in regard to its value at the basal frequency of 4 Hz compared to those of control, as we demonstrated before [51, 52]. None of the drug treatments were able to reverse this negative FFR in the T4-treated mice (Fig 2A). The effect of β-adrenergic stimulation was assessed by a concentration–response curve with isoproterenol (10^−9^–10^−6^ mol/l) at a baseline stimulation frequency of 4 Hz. Two-way ANOVA revealed that at least one of the drugs/isoproterenol concentrations differs from the others with respect to relative tension change (p = 0.000). One-way ANOVA showed that under full β-adrenergic stimulation (1 μmol/l isoproterenol), muscles from T4-treated mice exhibited significantly lowered responses (p < 0.05) versus those from control mice as we showed before [51, 52]. Nevertheless, none of the drug treatments were able to recover this blunted isoproterenol response in the T4-treated mice (Fig 2B). Blunted responses in the T4-treated mice could be possibly due to the development of arrhythmia. The % of muscles that exhibited arrhythmic behavior varied based on isoproterenol concentration, as shown in Fig 2C. At full μ-adrenergic stimulation (1 μmol/l isoproterenol): 13 out of 15 muscles from T4 group (87%), 8 out of 10 muscles from DMSO group (80%), 6 out of 9 muscles from sorafenib group (67%), 6 out of 10 muscles from tadalafil_IP_ group (60%), 5 out of 8 muscles from tadalafil_Or_ group (63%), 5 out of 8 muscles from CMC group (63%), 6 out of 7 muscles from macitentan_LD_ group (86%) and 7 out of 7 muscles from macitentan_HD_ group (100%) showed arrhythmic behavior versus 2 of 12 muscles in control mice (17%) (Fig 2C).

## Discussion

The goal of the current study was to test the hypothesis that multikinase inhibitor, sorafenib, PDE-5 inhibitor, tadalafil, and dual endothelin-1 receptor blocker, macitentan, could be potential therapies for cardiac remodeling, RV contractile abnormalities and/or LV systolic dysfunction in experimental hyperthyroidism.

Consistent with published data [51, 52, 58], T4 increased blood pressure of mice in the current study. However, sorafenib, tadalafil, and macitentan at different doses could not decrease such increase following T4 treatment. Typically, VEGF is a known vasodilator and its inhibition causes elevation of blood pressure, a well-known side effect of this class of anticancer drugs [59]. Sorafenib has been reported to increase blood pressure in both animals [60] and humans [61]. In this study, sorafenib could not decrease T4-induced hypertension in mice; however, it did not cause any further increase in the blood pressure of these mice. Alternatively, PDE-5 inhibitors are known to cause a moderate transient decline in blood pressure, which reveals the existence of PDE-5 in vascular smooth muscle cells and the NO/cGMP pathway role in the systemic blood pressure regulation [62]. Yet, some reports showed that PDE-5 inhibitors decrease blood pressure both in animals [63] and humans [64] even as other studies showed no effect in both species [65, 66], respectively. Dishy et al. [66] have attributed these inconsistencies to the multifactorial causes/mechanisms of decreased NO and subsequent endothelial dysfunction, with the possibility of being improved by PDE-5 inhibitors in some conditions but not in the others as we demonstrated here. Similarly, inconsistent results have been reported about the effects of endothelin-1 receptor blockers on blood pressure, where macitentan and bosentan were shown to decrease the systemic blood pressure in some animal models [41, 49, 59] but not in others [60–62]. In agreement with these latter reports, macitentan could not significantly decrease the blood pressure of the T4-treated mice in this study.

The development of cardiac hypertrophy following T4 treatment is well documented [31–33, 50–52, 58], and has been repeatedly linked to LV dysfunction [2, 31–33, 51, 52] as well as marked RV contractile abnormalities, as we previously described [51, 52]. Consistent with these data, we showed here that T4 treatment resulted in cardiac hypertrophy as indicated by increased heart weigh, heart weight/body weight ratio, LV mass and LV mass/body weight ratio of mice. Besides, it caused considerable in*-vivo* LV contractile dysfunction as evident by significantly reduced LV ejection fraction (EF) and fractional shortening (FS). Similarly, isolated RV papillary muscles from T4-treated mice demonstrated the hallmarks of hypertrophied and dysfunctional hearts, which are negative FFR and blunted β-adrenergic response along with decreased contraction/relaxation times. However, sorafenib, tadalafil or macitentan at all studied doses were not able to significantly attenuate the T4-induced abnormalities, including the cardiac hypertrophy, *in-vivo* LV dysfunction or *ex-vivo* RV contractile defects.

It has been proposed that combined inhibition of tyrosine and serine/threonine kinases by drugs such as sorafenib (10 mg/kg/day) may offer an option to treat PAH and associated RV remodeling in different preclinical models [8, 10]. Yet, numerous kinase inhibitors have been linked to marked cardiovascular toxicities, including contractile dysfunction and heart failure [53]. Incidentally, sorafenib (30–40 mg/kg/day) has been reported to induce myocyte necrosis, even in the absence of cardiac injury, and to dramatically increase mortality when administered in the presence of myocardial infarction [53]. Conversely, a retrospective analysis of the impact of sorafenib on cardiac function in patients with thyroid cancer revealed that cardiac toxicities did not raise a concern but, cardiac monitoring is still recommended [67]. In the current study, sorafenib could not reverse T4-induced cardiac defects, but, it also did not result in any major deterioration of cardiac hypertrophy or cardiac dysfunction compared to T4-treated mice. The increase in heart weight/body weight ratio in sorafenib-treated mice is clearly attributed to the significantly reduced body weights in these mice. Reduced body weight following sorafenib treatment was evident both in animals [53] and human patients [68]. It is worth mentioning that beneficial effects of sorafenib on RV remodeling/dysfunction was attributed to the inhibition of Raf/MEK/ERK pathway, a downstream of Ras signaling [10]. The ineffectiveness of sorafenib in this study may be in close agreement with our previous report showing that Ras signaling has no major role in the T4-induced cardiomyopathy in our model [51].

Also, PDE-5 inhibitors were found to exhibit cardioprotective effects against cardiac remodeling, cardiac injury and LV [19–28] as well as RV failure both in animals [29] and humans [30]. However, they failed to attain the same efficacy in other studies [69, 70]. In harmony with these latter studies, our current data showed that tadalafil could not attenuate cardiac hypertrophy or reverse cardiac contractile defects in the T4-treated mice. Of note, the most commonly reported pathway involved in PDE-5 inhibitors-mediated cardioprotection is the NO/cGMP/protein kinase (PK) G pathway. Nevertheless, in T4-induced hypertrophy, cGMP-PDE (PDE-5) activity was shown to be not affected [71], and cGMP levels were either not altered [71, 72] or decreased [73]. Similarly, PKG was found decreased [71] or unchanged in T4-induced hypertrophy [74]. Other studies revealed that the importance of this pathway (cGMP/PKG) was diminished [75] and that cGMP exerted negative functional effects in T4-induced hypertrophic myocytes independent of PKG [76]. Interestingly, nitroprusside, an activator of guanylate cyclase that increases cGMP, when topically applied on the hearts of hyperthyroid anesthetized open-chest rabbits increased the cGMP levels without changing the hypertrophy or hypertension in these rabbits [72]. Again, these observations support the negative outcomes of tadalafil in this study. In contrast, cardioprotective effects of PDE-5 inhibitors have been evident through many other signaling pathways, which are also common causative factors of T4-induced cardiac hypertrophy/dysfunction, such as oxidative stress, cardiomyocyte apoptosis, PI3K/Akt and ERK ½ [16, 17, 20, 31–34]. However, the lack of cardioprotective effects in this study excludes the possibility of these pathways from being affected by tadalafil.

Likewise, numerous studies have shown that ET-1 is a key player in the development of cardiac remodeling, LV as well as RV failure in both animals and humans [36–45]. Still, other studies have reported the opposite in both species [77–82]. In line with the latter studies, the dual ET-1 receptor blocker, macitentan, was not able to inhibit the T4-cardiac hypertrophy or to improve the cardiac dysfunction under *in-vivo* as well as *ex-vivo* settings in the current study. This may indicate that ET-1 has no major role in the T4-induced cardiomyopathy. Conversely, previous reports have shown that ET-1 contributes to cardiac hypertrophy and increased susceptibility to ischemia/reperfusion-induced ventricular fibrillation in the hyperthyroid myocardium [46–49]. Shohet et. al. [46] reported that mice with cardiomyocyte-specific disruption of the ET-1 gene are resistant to hyperthyroid cardiac hypertrophy. In addition, Tang et. al. [47] have demonstrated that a dual ET-1 receptor blocker, dajisentan (CPU0213), decreased the T4-induced cardiac hypertrophy in rats. The same group revealed that ET-1 is a key player in the increased susceptibility to ischemia/reperfusion-induced ventricular fibrillation in these rat hearts following T4 treatment [48, 49]. However, it is clear that there are apparent differences between these latter animal models and the animal model used in this study. For instance, in the study of Shohet et. al. [46], T3 treatment (1 mg/kg/I.P) in wild-type littermates of the genetically modified mice (3–4 months) for 3 weeks resulted in cardiac hypertrophy and preserved LVEF. In this study, however, T4 treatment (0.5 mg/kg/I.P) in mice (7–9 months) for 2 weeks resulted in cardiac hypertrophy and significantly reduced LVEF and LVFS. Very close to these discrepancies, ET-1 has been shown to be involved in the cardiac hypertrophy induced by angiotensin-II [83] or sympathomimetics such as norepinephrine mainly through ET_A_ receptor [84]. However, data from cardiomyocyte-specific ET_A_ receptor knockout mice have shown that ET_A_ receptors are not necessary for either baseline cardiac function or stress-induced cardiac response to angiotensin-II or another sympathomimetic drug, isoproterenol [79]. Also, ET-1 has been shown to have a role in the angiotensin-II-induced cardiac hypertrophy in mice with vascular endothelial cell specific ET-1 deficiency [85]. Nonetheless, another study showed that it has no role in the cardiac hypertrophy induced by transaortic constriction in the same mice [80]. Thus, it has been concluded that ET-1 stimulates several cellular responses which may be conflicting based on the situations [80].

In the present study, HR has been assessed by ECG, tail cuff and echocardiography. In contrast to ECG and tail cuff analyses, echocardiography showed a significantly higher HR in the T4-treated mice compared to control. However, in echocardiography, one should count the impact of anesthesia on HR, since different anesthetic agents may cause various degrees of suppression in mouse autonomic and HR [86]. Additionally, tail cuff and echocardiography allow for the indirect HR assessment once the number of cycles of either arterial pressure waves or cardiac contractions in a specified time is calculated, respectively. Since these methods can only detect variations in cycle length of succeeded cardiac contractions and cannot distinguish ectopic beats from sinus beats, the ECG is considered as the “gold standard” for the detection of heart rate [86]. Here, ECG analysis demonstrated similar values for HR of both control and T4-treated mice, which fall into the range of normal murine HR of 500–700 BPM, as reported before [86]. Although T4-induced tachycardia is one of the most commonly used diagnostic parameters for detection of hyperthyroidism HR may not always be a good predictor of hyperthyroidism, especially when the disease has been present for prolonged time and the hearts progressed to heart failure [2].

## Conclusions

We show here for the first time that multikinase (raf1/b, VEGFR, PDGFR), PDE-5, and endothelin-1 pathways have no major role in T4-induced hemodynamic changes and cardiac abnormalities. In particular, our data show that the involvement of endothelin-1 pathway in T4-induced cardiac hypertrophy/dysfunction seems to be model-dependent and should be carefully interpreted.

## Supporting Information

S1 Table Spread sheet of blood pressure data. (XLSX)

S2 Table Spread sheet of ECG data. (XLSX)

S3 Table Spread sheet of echocardiography data. (XLSX)

S4 Table Spread sheet of heart rate data. (XLSX)

S5 Table Spread sheet of morphological data. (XLSX)

S6 Table Spread sheet of papillary muscle data. (XLSX)
